# Optimizing surgical efficiency: a critical appraisal of machine learning in forecasting operative duration for metabolic and bariatric procedures

**DOI:** 10.1097/JS9.0000000000001262

**Published:** 2024-03-05

**Authors:** Cheng Guo, Qinghui Cai, Kai Liu

**Affiliations:** a Comprehensive Pediatrics and Pulmonary and Critical Care Medicine, Kunming Children’s Hospital Kunming, Yunnan Province; b Qionghai People’s Hospital, Qionghai, Hainan Province, People’s Republic of China

*Dear Editor*,

Your research paper ‘Predicting operative time for metabolic and bariatric surgery using machine learning models: a retrospective observational study’ provides a metabolic and bariatric surgery (MBS) operative time prediction in depth^1^. This study utilizes machine learning (ML) techniques, specifically XGBoost models, to predict surgery times for metabolic and bariatric surgery, an approach that shows potential for improving operating room efficiency and patient care. The authors demonstrated technical innovation by using a variety of ML models, particularly XGBoost, to make predictions about surgical time. However, in this article, we note some key limitations that warrant further exploration and improvement.

Firstly, it is recommended to enhance the interpretability of the model, although the predictive accuracy of the model is important, improving the interpretability of the model will help clinical applications and future studies could explore how to use interpretable AI to achieve this^2^. Secondly, the effect of surgeon skill and experience needs to be considered; surgeon skill and experience have a significant effect on operative time^3^, and incorporating these factors into the model may improve prediction accuracy, as well as the type of anesthesia and preoperative preparation time, which were not included in the study, and which have an effect on total operating room occupancy time^4^. It is recommended that future studies consider these factors to more accurately predict overall OR occupancy time. Finally, the study did not address specific technical details of surgery, such as management of comorbidities and choice of surgical route, which may affect operative time^5^. Future studies may consider adding these variables to more fully predict surgical time.

Overall, this study provides valuable insights into surgical time prediction, and if these findings can be applied in practice, it could optimize OR scheduling and management, but further refinement and expansion is needed to increase its value in clinical practice.

## Ethical approval

Not applicable.

## Consent

Not applicable.

## Sources of funding

Unfunded support.

## Author contribution

C.G. and Q.C.: writing – original draft; K.L.: writing – reviewing and editing. All authors contributed to the article and approved the submitted version.

## Conflicts of interest disclosure

The authors declare no conflicts of interest.

## Research registration unique identifying number (UIN)

1. Name of the registry: not applicable.
2. Unique identifying number or registration ID: not applicable.
3. Hyperlink to your specific registration (must be publicly accessible and will be checked): not applicable.

## Guarantor

Kai Liu.

## Data availability statement

The data underlying this article will be shared by the corresponding author on reasonable request. No data were generated during the writing of this study.

## Provenance and peer review

Not applicable.
